# Case report: Otitis media with subsequent mastoiditis and cerebral herniation in a patient with Arnold chiari malformation

**DOI:** 10.3389/fped.2022.1013300

**Published:** 2023-01-23

**Authors:** Oskar Feussner, Roland Haase, Jan Baier

**Affiliations:** Department for Operative and Nonoperative Pediatrics and Adolescent Medicine, Section for Neonatology and Pediatric Critical Care, University Hospital, Halle, Germany

**Keywords:** mastoiditis, otitis media, arnold-chiari-malformation, sudden death, brain absces

## Abstract

We present the case of a 13-year-old boy who unexpectedly needed to be resuscitated at home after an assumed uncomplicated otitis media. Imaging at our clinic showed mastoiditis and a cystoid mass in the left cerebellopontine angle compressing the brainstem, as well as an Arnold-Chiari-Malformation. Both the laboratory examination of cerebrospinal fluid (CSF) and surgical biopsy with pathological evaluation of the mastoid supported the inflammatory etiology of the mass. Microbiologically, Streptococcus intermedius was detected in the blood culture and CSF. Due to brain death, which most likely already existed preclinically, the organs were released for donation during the course. Our case demonstrates a very rare lethal complication of acute otitis media on the basis of a cerebral malformation and emphasizes the need to stay alert when patients complain of symptoms after assumed resolution.

## Introduction

Mastoiditis, despite being the most common severe complication of acute otitis media (AOM), is a nowadays rare condition (1, 2). However, since it is a potentially life-threatening disease, it is of great importance to recognize symptoms in time. Depending on age, these include predominantly a protruding ear, retroauricular erythema, swelling or pain, ear discharge, fever, and a deteriorated general condition (3). Particularly challenging are less symptomatic or even asymptomatic courses, which have been described especially in cases in which AOM was treated with an antibiotic (4). Diagnostics include a medical history, the physical examination and laboratory diagnostics of the blood. However, there is still no consensus on the need and timing of computer tomography (CT). For treatment, research demonstrated that in uncomplicated cases, conservative management (antibiotic treatment with or without myringotomy and ventilation tubes) is an efficient first-line treatment. However, mastoidectomy should be performed in case of failure of conservative therapy after 48–72 h or in the case of complications, e.g., epidural or subdural abscess, facial paralysis, sinus thrombosis or brain abscess. As a result, with appropriate therapy, the prognosis is generally favorable with few long-time complications (5).

## Case description

**Figure 1:**
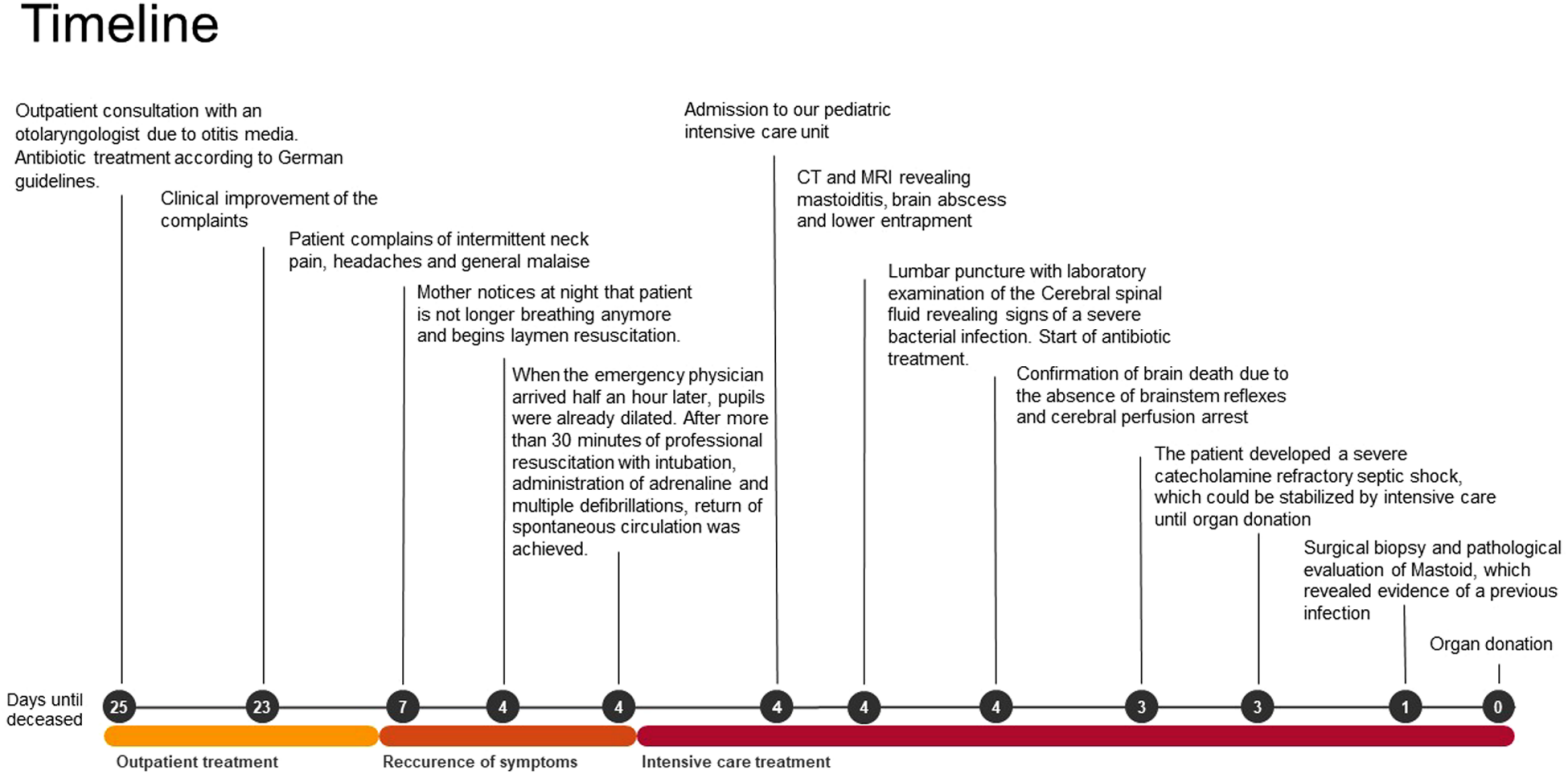
Timeline with chronological depiction of events from outpatient diagnosis of otitis media to organ donation.

**Figure 2:**
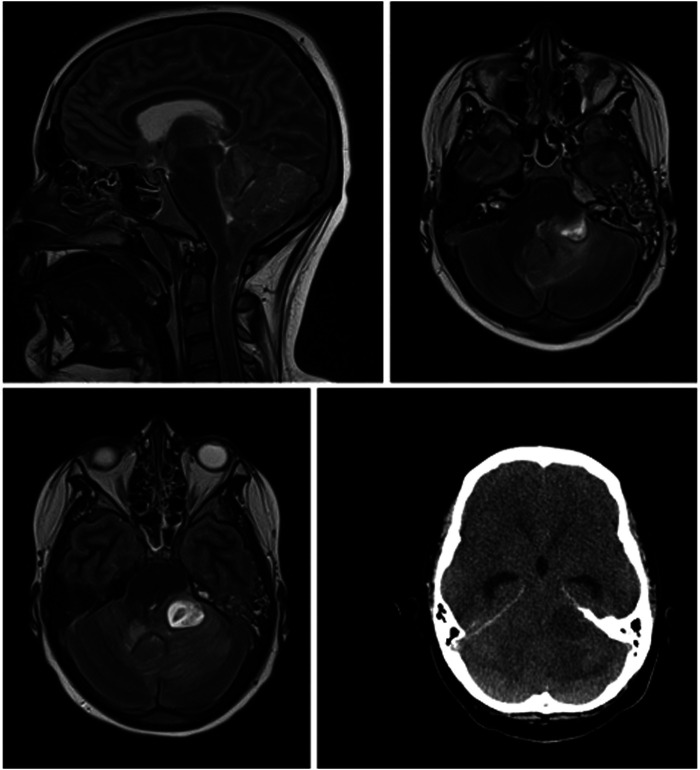
cMRI and cCT show lower entrapment most likely caused by caudal ectopia of the cerebellar tonsils and a cystoid mass in the left cerebellopontine angle compressing the brainstem, with prominent surrounding cerebellar edema and inflammatory changes in the area of the petrous bone and the left mastoid.

We present the case of a 13-year-old boy with obesity and hypertension who unexpectedly needed to be resuscitated at home by his mother (for the timeline see Figure 1). When the emergency physician arrived half an hour later, pupils were already dilated. After more than 30 minutes of professional resuscitation with intubation, administration of adrenaline and multiple defibrillations, spontaneous circulation was achieved. Intubated and ventilated, the patient was then admitted to our pediatric intensive care unit (PICU), where we performed a cranial computed tomography (CT) and, in the course, a cranial magnetic resonance imaging (MRI), in which the clinical suspicion of a lower entrapment was confirmed. The most likely cause appeared to be the caudal ectopia of the cerebellar tonsils and a cystoid mass in the left cerebellopontine angle compressing the brainstem, with prominent surrounding cerebellar edema and inflammatory changes in the area of the petrous bone and the left mastoid (see Figure 2). Laboratory results on admission are shown in Table 1.

**Table 1:** Laboratory results on admission.

Blood sample	Liquor sample
Blood count: leucocytes 13.1 Gpt/L, haemoglobin 7.7 mmol/L, thrombocytes 402 Gpt/L	Cell count: leucocytes 5,396 Mpt/L, 99% Neutrophils, erythrocytes 1,196 Mpt/L
Coagulation tests: INR 1.26, PTT 27 s, fibrinogen 5.28 g/L, AT III 120%	Protein >6 g/L, Glucose <0,1 mmol/L, lactate 25 mmol/L
Further clinical chemical results: creatinine 74 µmol/L, Urea 5.5 mmol/L, ASAT 1.49 µcat/L, ALAT 2.30 µcat/L, GGT 1.98 µcat/L, CK 1.88 µcat/L, CK-MB 0.6 µcat/L, myoglobin 24 µg/L, troponin T 321 ng/L, NT-pro BNP 2300 ng/L, CRP 28,4 mg/L	

A conversation with the mother revealed, that three weeks earlier, the patient was diagnosed with Acute Otitis media (AOM) and treated with oral antibiotics according to German guidelines. As a result, there had been a clinical improvement without symptom progression or fever. However, three days before admission to our PICU, the patient complained of intermittent neck pain and headache and was not feeling well. Because of this, he had slept in his mother's bed where he had been talking while sleeping, then suddenly paused and stopped breathing. Other symptoms in relation to possible neurological, oncological or infectious diseases were negated. The patient's medical history only revealed arterial hypertension treated with metoprolol, and a pronounced obesity of 110 kg body weight. In addition, the patient had repeatedly complained of headaches and decreased physical resilience for the last eight weeks. The physical examination on our PICU showed a mildly inflamed left tympanic membrane, consistent with an otitis media that had subsided a few weeks earlier. The patient was completely vaccinated according to the recommendations of the Robert Koch institute including two vaccinations with Comirnaty.

In the absence of brainstem reflexes and cerebral perfusion arrest, we confirmed brain death one day after admission according to the guidelines of the German medical association. We discussed the possibility of organ donation with the parents, who consented.

For differential diagnostic clarification of the etiology of the mass, we performed a lumbar puncture. Laboratory examination of the Cerebral spinal fluid (CSF) revealed signs of a severe bacterial infection, which supported the inflammatory etiology of the mass. Microbiologically, Streptococcus intermedius was detected both in the blood culture and in the CSF, which showed no resistances against standard antibiotics. Despite extensive diagnostics, there was no evidence of an acute viral infection. Myocarditis could be excluded by further investigations. Antibiotic treatment was started right after the diagnostic work-up.

In the meantime, the patient developed a severe catecholamine refractory septic shock, which could be stabilized by intensive care until organ donation. To rule out a malignant process of the left mastoid before organ transplantation, we performed surgical biopsy and pathological evaluation, which revealed evidence of a previous infection of the mastoid with fibrosed mucosa. A neoplastic process could be excluded. After clarification of the microbiological etiology of the cerebellar abscess, organ donation could proceed without complications.

## Discussion

This case illustrates the rare but life-threatening presentation of mastoiditis with subsequent subdural empyema and cerebral herniation after AOM.

With a cumulative prevalence of more than 60% by the age of seven, AOM is not only a highly common disease (6) but it is also the most prevalent reason for antibiotic therapy in young children (7). Despite the high incidence, severe complications are rare (8). These include, with decreasing probability, mastoiditis, sub-periosteal abscess, facial nerve palsy, epidural abscess, sigmoid sinus and internal jugular vein thrombosis. Other complications such as elevated intracranial pressure, cerebral stroke and suppurative meningitis are rare and have been described only in single cases (1). Mastoiditis, as the most common complication, was observed in the United Kingdom in 1.8 per 10,000 AOM episodes in which antibiotics were administered and in 3.8 per 10,000 AOM episodes in which no antibiotic was administered (9), whereas a meta-analysis described 23.7 cases of mastoiditis per 10,000 episodes of AOM (10). Depending on the investigated country and time period, the incidence rate of Mastoiditis is reported to be 1.2–4.8/100,000 person-years (2, 11).

Regarding the dynamics of incidence in the last years and decades, there are conflicting findings. Several studies indicate an increase in incidence and an increase in surgical intervention and attribute this to the rising number of antibiotic resistances (12, 13). On the other hand, numerous studies report a constant or even decreasing incidence (2, 9, 11, 14). It should be noted, however, that an increase in the complication rate and the need for surgical intervention was observed here as well (14).

However, precise data on changes in incidence over the last two decades is important, as a possible rise in incidence has been associated with a more restrictive usage of antibiotics (2). This is supported by the fact, that Thompson and colleagues observed in their cohort study that antibiotic treatment for AOM reduced the risk of future mastoiditis by 50% (9). Arguing against the more liberal use of antibiotics in AOM, other studies have not only observed no effect of antibiotic administration on the risk of mastoiditis (15) but in some cases prior antibiotic administration was even associated with an increased risk for the need for surgical intervention (16). In addition, given the rarity of mastoiditis, the number needed to treat with an antibiotic to prevent one case of mastoiditis is estimated to be between 2,500 and 4,381 (9, 17). Here, however, the benefits bear no relation to the financial costs, the possible side effects and the development of bacterial resistance. Furthermore, our case supports the studies showing that despite antibiotic treatment, complete prevention is not provided and prior antibiotic administration may even mask mastoiditis (4, 18). In our case, however, it should be noted that the potential standard dosage of the antibiotic in an obese patient may have been a contributing factor to treatment failure.

The most common pathogens of mastoiditis include Streptococcus Pneumoniae, Pseudomonas aeruginosa, Streptococcus Pyogenes group A, Staphylococcus aureus, and Haemophilus influenzae (1, 11, 19). In addition, Fusobacterium Necrophorum has been detected more frequently in mastoiditis in recent years (14, 20), particularly in cases previously treated with antibiotics or where complications occurred and surgery was required (14, 19). Concerningly, a rising number of resistant infections have been found in mastoiditis in recent years as well (1, 13).

Streptococcus intermedius, a commensal microorganism of the oral flora and the bacterium we consider to be the most likely causative, does not appear to be a typical pathogen of mastoiditis. It does, however, appear to be a common pathogen associated with brain abscesses (21). There are case reports describing brain abscesses in children after mastoiditis caused by Streptococcus intermedius (22). Pathophysiologically, the brain abscess formation occurs after tissue damage, which is followed by bacterial colonization and subsequent tissue liquefaction and pus formation due to hyaluronidase activity of Streptococcus intermedius (21).

It is also worth mentioning that on MRI, in addition to the mass compressing the brainstem, there was a descent of the cerebellar tonsils, indicating a previously unknown Arnold-Chiari malformation Type 1. This as a rare craniovertebral junction malformation with caudal ectopia of the cerebellar tonsils through the Foramen Magnum that can lead to slowly progressive symptoms like headaches, neck pain, motor deficits, cranial nerve palsy, oropharyngeal dysfunctions and sleep disorders (23, 24). Epidemiologic data are scarce and with wide discrepancies. For instance, two retrospective analyses of MRI images reported incidences of 0.77 and 3.6%, respectively (25, 26). Matching our case, the disease was observed more frequently in children and young adults and the male sex (23). However, there is a minority of asymptomatic patients with an acute fatal onset (27, 28). Although, comparable to the case described by Stephany et al. the patient headaches, which he had described for several weeks can retrospectively be considered as a possible prodrome (29). Due to the ectopic position of the cerebellar tonsils through the foramen magnum into the spinal canal and the thus chronically constricted brainstem, patients with Arnold-Chiari malformation are particularly susceptible to cerebellar tonsillar impaction. Minor trauma, or as in our case, an intracerebral mass, can promptly decompensate the vulnerable system. Pathophysiologically, both direct mechanical entrapment of brain tissue and compression of cerebral vessels with subsequent ischemia can impair the function of the respiratory and circulator as well as the ascending reticular activating system with direct catastrophic consequences (23). In our case, this might have contributed to the rapid clinical deterioration and ultimately cardiovascular failure.

Although the coincidence of an Arnold-Chiari malformation and mastoiditis is rare, this case illustrates that even nowadays otitis media can have a severe and sometimes lethal outcome. Especially, recurring complaints such as headache, fever or neck pain should always raise the suspicion of unsuccessful treatment and require a conscientious investigation to initiate the appropriate diagnostics and subsequent therapy in time. To prevent a fatal course, as in our case, it is, therefore, crucial to educate the patient and parents about the need for immediate reappearance in the event of the above-mentioned complaints, some of which may occur after a latency of 8–12 weeks. Finally, this case also highlights that antibiotic therapy never reliably protects against serious complications and, in the worst case, can even lead to a delay in diagnosis because the treating physician is under a false sense of security.

## Data Availability

The original contributions presented in the study are included in the article/Supplementary Material, further inquiries can be directed to the corresponding author/s.
